# Draft Genome Sequence of a *Mycobacterium avium* Complex Isolate from a Broadbill Bird

**DOI:** 10.1128/genomeA.01268-13

**Published:** 2014-01-30

**Authors:** John P. Bannantine, Darrell O. Bayles, Suelee Robbe-Austerman, Angela M. Burrell, Judith R. Stabel

**Affiliations:** a National Animal Disease Center-USDA-ARS, Ames, Iowa, USA; b National Veterinary Services Laboratory-USDA-APHIS, Ames, Iowa, USA; c Life Technologies, Austin, Texas, USA

## Abstract

We report the draft genome sequence of a *Mycobacterium avium* complex isolate. This isolate has an estimated genome size of 5.1 Mb with an average GC content of 68.9% and is predicted to carry 4,497 protein-encoding genes and 317 pseudogenes.

## GENOME ANNOUNCEMENT

*Mycobacterium avium* complex (MAC) organisms cause opportunistic infections in humans, yet their epidemiology remains poorly understood. They are slowly growing environmental and animal-associated mycobacteria that have little notoriety except for the strains that cause disseminated infections in HIV-infected humans (1). Most MAC organisms are classified taxonomically as a single species, *M. avium*, which is divided into at least four subspecies, *M. avium* subsp. *avium*, *M. avium* subsp. *hominissuis*, *M. avium* subsp. *paratuberculosis*, and *M. avium* subsp. *silvaticum* (2). The only other species in this group is *M. intracellulare*. Genotyping of this diverse bacterial group has been achieved using intergenic spacers (3) and *rpoB* sequence analysis (4, 5).

The genome sequences of *M. avium* subsp. *hominissuis* strain 104 and *M. avium* subsp. *avium* ATCC 25291 have been made publically available, but neither has yet been formally published. Strain 104 was isolated from an immunocompromised human and ATCC 25291 was obtained from a bird. Here we present the draft genome sequence of another *M. avium* subspecies. This isolate was obtained from a broadbill bird (family Eurylaimidae; species not specified) from a Texas zoo in 2005. Analysis of the *rpoB* gene sequence using the method of Ben Salah et al. (4) shows that this isolate clusters with *M. avium* subsp. *hominissuis*.

Purified genomic DNA obtained from this isolate was subjected to whole-genome shotgun sequencing using the Ion PGM sequencing system (Ion Torrent) with 200-bp read chemistry (Life Technologies). Sequence coverage was 81.0×. A *de novo* assembly of the ~5.15-Mbp sequence using MIRA assembly software version 3.2.0 yielded 190 contigs. The sequence was annotated with the NCBI Prokaryotic Genomes Automatic Annotation Pipeline (http://www.ncbi.nlm.nih.gov/genome/annotation_prok/). A total of 5 rRNA operons and 46 tRNA genes were identified.

### Nucleotide sequence accession numbers.

This whole-genome shotgun project was deposited at DDBJ/EMBL/GenBank under the accession number AYLW00000000. The version described in this paper is AYLW01000000.

## References

[1] Inderlied CB Kemper CA Bermudez LE 1993 The *Mycobacterium avium* complex. Clin. Microbiol. Rev. 6(3):266–310 8358707 10.1128/cmr.6.3.266 PMC358286

[2] Alexander DC Turenne CY Behr MA 2009 Insertion and deletion events that define the pathogen *Mycobacterium avium* subsp. *paratuberculosis*. J. Bacteriol. 191:1018–1025. 10.1128/JB.01340-08 19028885 PMC2632067

[3] Cayrou C Turenne C Behr MA Drancourt M 2010 Genotyping of *Mycobacterium avium* complex organisms using multispacer sequence typing. Microbiology 156 (Pt 3):687–694. 10.1099/mic.0.033522-0 19926652

[4] Ben Salah I Adékambi T Raoult D Drancourt M 2008 *rpoB* sequence-based identification of *Mycobacterium avium* complex species. Microbiology 154 (Pt 2):3715–3723. 10.1099/mic.0.2008/020164-0 19047739

[5] Higgins J Camp P Farrell D Bravo D Pate M Robbe-Austerman S 2011 Identification of *Mycobacterium* spp. of veterinary importance using rpoB gene sequencing. BMC Vet. Res. 7:77. 10.1186/1746-6148-7-77 22118247 PMC3251535

